# Hyperuricemia is associated with more cardiometabolic risk factors in hypertensive younger Chinese adults than in elderly

**DOI:** 10.3389/fcvm.2023.1133724

**Published:** 2023-03-17

**Authors:** Xiaofeng Su, Jing Liu, Ningling Sun, Yong Huo, the SUCCESS Investigation Group

**Affiliations:** ^1^ Department of Hypertension, Peking University People’s Hospital, Beijing, China; ^2^ Cardio-Metabolic Laboratory, Peking University People's Hospital, Beijing, China; ^3^ Department of Cardiology, Peking University First Hospital, Beijing, China

**Keywords:** hyperuricemia, cardiometabolic risk factors, hypertension, age-related, cross-section study

## Abstract

**Background:**

Numerous studies have shown that hyperuricemia (HUA) is associated with cardiovascular and renal outcomes, but few studies specifically explored the effect of age on this relationship. Therefore, our study aimed to explore the relationship between HUA and other cardiometabolic risk factors in different age groups.

**Methods:**

This cross-section study used the data from Survey on uric acid in Chinese subjects with essential hypertension (SUCCESS). We performed multivariate logistic regressions in different age groups.

**Results:**

After adjusting for potential confounders, among young and middle-aged adults less than 60, HUA was associated with higher body mass index (BMI, adjusted OR = 1.114, 95% CI: 1.057–1.174), higher fasting blood glucose (FBG, adjusted OR = 1.099, 95% CI: 1.003–1.205), triglycerides (TG, adjusted OR = 1.425, 95% CI: 1.247–1.629), higher low-density lipoprotein cholesterol (LDL-C, adjusted OR = 1.171, 95% CI: 1.025–1.337), and lower estimated glomerular filtration rate (eGFR, adjusted OR = 0.992, 95% CI: 0.988–0.996). Among elderly adults 60 years or older, HUA was associated with higher SBP (adjusted OR = 1.024, 95% CI: 1.005–1.042), higher TG (adjusted OR = 1.716, 95% CI: 1.466–2.009), and higher LDL-C (adjusted OR = 1.595, 95% CI: 1.366–1.863).

**Conclusion:**

HUA is associated with more cardiometabolic risk factors in younger adults with hypertension (HT). Comprehensive management of HT with HUA is needed in clinical settings.

## Introduction

Hyperuricemia (HUA) is a metabolic disorder caused by abnormal purine catabolism and urate excretion *via* urate transporters (1, 2). Numerous epidemiological studies have explored the association between HUA and cardiovascular and renal outcomes (3, 4). It is suggested that HUA is associated with hypertension (HT) (5), cardiovascular death (6), diabetes (7), chronic kidney disease (CKD) (8) and stroke (9).

It is noteworthy that previous studies have shown the age-differential relationship between HUA and HT (10, 11) or CKD (12), which provides a new idea for studying the mechanism of uric acid (UA)-related metabolic disorders and the individualized management of HUA.

However, most studies adjusted age as a covariate in the analysis, and few studies specifically explored the effect of age on the relationship between elevated UA and other cardiometabolic risk factors, such as dyslipidemia, obesity, and glucose metabolism disorders in hypertensive population. Therefore, we aimed to explore the relationship between HUA and other cardiometabolic risk factors in different age groups.

## Methods

### Study population

The data analyzed in this article came from SUCCESS. It was a nationwide cross-sectional study, which included adult hypertensive patients from 17 provinces and cities across. Patients who took more than one oral antihypertensive drug and those who had gout or received urate-lowering treatment were excluded. Details of the research proposal have been published elsewhere (13).

In this study, only individuals from Beijing (*n* = 4,126) were included, and individuals with error values (*n* = 90), missing fundamental values (*n* = 49), and outliers (*n* = 64) were excluded. Finally, 3,923 individuals were analyzed. They were divided into two groups based on age, the young and middle-aged group (under 60 years) and the elderly group (60 years or older).

All participants provided written informed consent, and the Peking University First Hospital Central Institutional Review Board approved the study protocol.

### Questionnaire data and physical examination

Each participant's clinical information, including age, sex, education level, and medication history, was collected using standard questionnaires by the trained physicians. The education level was divided into junior high school or below, senior high school, junior college, and undergraduate or above. Medication history included the use of antihypertensive agents and aspirin.

The physical examination information of participants was also measured and calculated by professional doctors in the clinic room, including height (cm), weight (kg), waist circumference (cm), body mass index (BMI, kg/m^2^), SBP (mmHg), and diastolic blood pressure (DBP, mmHg).

### Laboratory assays

All blood samples of the subjects were taken 8 h of fasting, and parameters such as FBG, total cholesterol (TC), LDL-C, high-density lipoprotein cholesterol (HDL-C), TG, UA, and serum creatinine (SCr) were obtained through routine biochemical tests. The eGFR was calculated according to the Cockcroft-Gault equation. This study defined HUA as uric acid of more than 420 µmol/L in men or 360 µmol/L in women. All individuals were divided into HUA and non-HUA groups.

### Statistical analysis

Continuous variables were described as mean ± standard deviation (SD), and categorical variables were described as quantity (frequency or percentage). The *t*-test and the Chi-square test were used to compare the variables between different groups (young and middle-aged group and elderly group, HUA group and non-HUA group). Then we conducted multivariate logistic regressions in different age groups, adjusting for age, sex, education level, aspirin use, and antihypertensive agents by including these factors into the regression equation at the same time. All the analysis was completed with IBM SPSS for windows, version 24.0, and statistical significance was defined as *p* value of <0.05.

## Results

### Characteristics of hypertensive adults

A total of 3,923 hypertensive adults were analyzed, including 2,240 adults in the young and middle-aged group and 1,683 adults in the elderly group. The demographic characteristics, medications and cardiometabolic profiles are shown in Tables 1, 2. The average age of the two groups was 50.3 and 68.3 years old, respectively.

**Table 1:** Demographic characteristics and medications of hypertensive adults in different age group.

Characteristic	18–59 years old	≥60 years old	*p* value
**Number**	2,240	1,683	
**Age (years)**	**(50.3 ± 6.0)**	**(68.3 ± 6.7)**	**<0** **.** **001**
**Gender**			**0.024**
Female	902 (40.3%)	738 (43.9%)	
Male	1,338 (59.7%)	945 (56.1%)	
**Education level**			**<0** **.** **001**
Junior high school or below	292 (13.0%)	591 (35.1%)	
Senior high school	450 (20.1%)	558 (33.2%)	
Junior college	740 (33.0%)	416 (24.7%)	
Undergraduate or above	758 (33.8%)	118 (7.0%)	
**Use of aspirin**			**<0** **.** **001**
No	1,736 (77.5%)	918 (54.5%)	
Yes	504 (22.5%)	765 (45.5%)	
**Use of antihypertensive agents**			**<0** **.** **001**
No	89 (4.0%)	28 (1.7%)	
ACEIs	294 (13.1%)	216 (12.8%)	
ARBs	992 (44.3%)	739 (43.9%)	
Beta blockers	764 (34.1%)	636 (37.8%)	
CCBs	72 (3.2%)	50 (3.0%)	
Diuretics	29 (1.3%)	14 (0.8%)	

**Figure 1:**
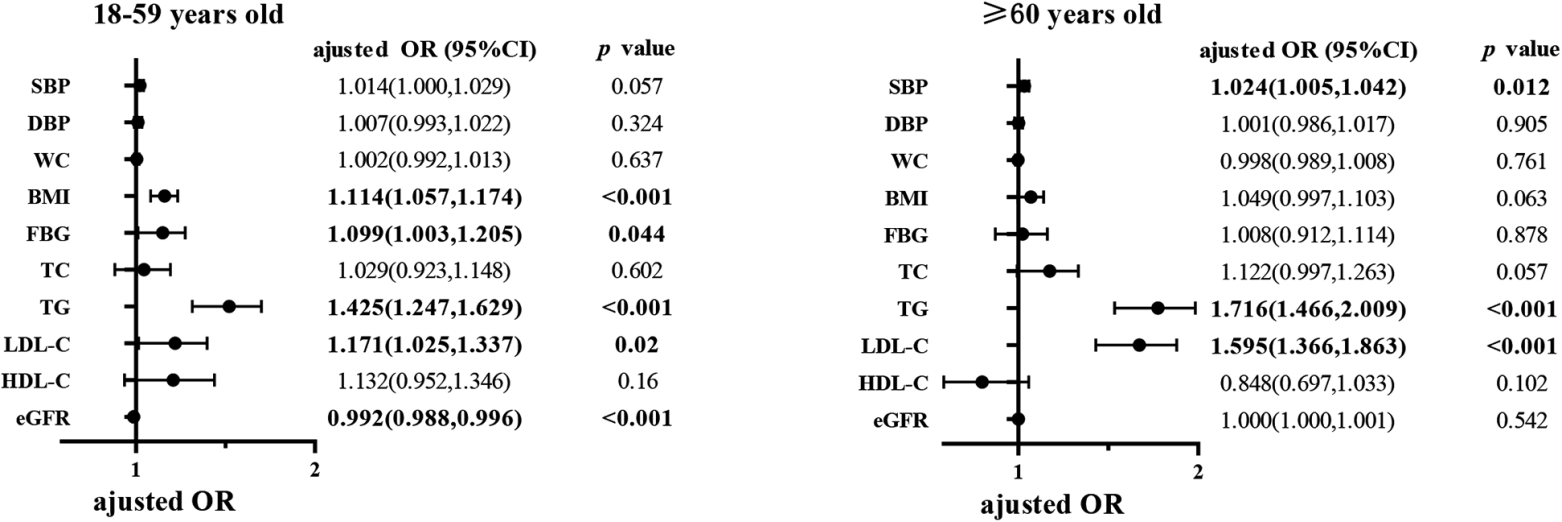
Multivariate logistic regression analysis of HUA in different age groups. The Odds ratios were adjusted by including age, sex, education level, use of aspirin, use of antihypertensive agents and all the above cardiometabolic factors into the regression equation at the same time. HUA, hyperuricemia; OR, odds ratio; CI, confidence interval; SBP, systolic blood pressure; DBP, diastolic blood pressure; WC, waist circumference; BMI, body mass index; FBG, fasting blood glucose; TC, total cholesterol; TG, triglycerides; LDL-C, low density lipoprotein cholesterol; HDL-C, high density lipoprotein cholesterol; eGFR, estimated glomerular filtration rate. The bold value represents that the *p* < 0.05, and its corresponding mean ± standard deviation in table 1-4 or adjusted OR (95%CI) in figure 1.

The bold value represents that the *p* < 0.05, and its corresponding mean ± standard deviation in table 1-4 or adjusted OR (95%CI) in figure 1.

Data are given as percentages or mean ± SD, unless otherwise indicated. ACEIs, angiotensin converting enzyme inhibitors; ARB, angiotensin receptor blockers; CCBs, calcium channel blockers.

**Table 2:** Cardiometabolic profiles of hypertensive adults in different age groups.

Characteristic	18–59 years old	≥60 years old	*p* value
SBP (mmHg)	**(151.9 ± 8.9)**	**(150.7 ± 7.7)**	**<0** **.** **001**
DBP (mmHg)	**(87.9 ± 8.1)**	**(86.2 ± 8.5)**	**<0** **.** **001**
WC (cm)	**(85.9 ± 13.4)**	**(89.3 ± 17.1)**	**<0** **.** **001**
BMI (kg/m^2^)	(24.9 ± 2.6)	(25.1 ± 2.7)	0.103
UA (µmol/L)	(323.4 ± 94.8)	(327.7 ± 103.8)	0.179
FBG (mmol/L)	**(5.7 ± 1.2)**	**(5.9 ± 1.3)**	**<0** **.** **001**
TC (mmol/L)	**(4.5 ± 1.1)**	**(4.6 ± 1.1)**	**<0** **.** **001**
TG (mmol/L)	**(1.6 ± 0.9)**	**(1.7 ± 0.9)**	**0.002**
LDL-C (mmol/L)	(2.4 ± 0.9)	(2.5 ± 1.0)	0.784
HDL-C (mmol/L)	(1.6 ± 0.7)	(1.6 ± 0.7)	0.617
SCr (µmol/L)	(71.6 ± 18.1)	(72.5 ± 24.8)	0.230
eGFR (ml/min/1.73m^2^)	(112.3 ± 48.1)	(102.4 ± 240.8)	0.097

The bold value represents that the *p* < 0.05, and its corresponding mean ± standard deviation in table 1-4 or adjusted OR (95%CI) in figure 1.

Data are given as percentages or mean ± SD, unless otherwise indicated. SBP, systolic blood pressure; DBP, diastolic blood pressure; WC, waist circumference; BMI, body mass index; UA, uric acid; FBG, fasting blood glucose; TC, total cholesterol; TG, triglycerides; LDL-C, low density lipoprotein cholesterol; HDL-C, high density lipoprotein cholesterol; SCr, serum creatinine; eGFR, estimated glomerular filtration rate.

Compared with the elderly group, the young and middle-aged group had a higher proportion of males, a higher education level (junior college and undergraduate or above), and a lower proportion used aspirin and antihypertensive agents.

Regarding cardiometabolic profiles, the levels of SBP and DBP in the young and middle-aged group were significantly higher than in the elderly group. However, waist circumference, FBG, TC, and TG levels were significantly lower. There was no significant difference in BMI, UA, LDL-C, HDL-C, SCr, and eGFR between the two groups.

### Characteristics of hypertensive adults with HUA and without HUA

The demographic characteristics, medications and cardiometabolic profiles of hypertensive adults with and without HUA in different age groups are presented in Tables 3, 4.

**Table 3:** Demographic characteristics and medications in hypertensive adults with and without HUA.

Characteristic	18–59 years old			≥60 years old		
	non-HUA	HUA	*p* value	non-HUA	HUA	*p* value
**Number**	1,821	419		1,280	403	
**Age (years)**	(50.3 ± 6.0)	(50.4 ± 6.4)	0.993	(68.3 ± 6.8)	(68.4 ± 6.3)	0.710
**Gender**			**0.001**			**<0.001**
Female	702 (38.6%)	200 (47.7%)		507 (39.6%)	231 (57.3%)	
Male	1,119 (61.4%)	219 (52.3%)		773 (60.4%)	172 (42.7%)	
Education level			0.473			0.320
Junior high school or below	234 (12.9%)	58 (13.8%)		442 (34.5%)	149 (37.0%)	
Senior high school	356 (19.5%)	94 (22.4%)		418 (32.7%)	140 (34.7%)	
Junior college	606 (33.3%)	134 (32.0%)		324 (25.3%)	92 (22.8%)	
Undergraduate or above	625 (34.3%)	133 (31.7%)		96 (7.5%)	22 (5.5%)	
Use of aspirin			**<0.001**			**<0.001**
No	1,446 (79.4%)	290 (69.2%)		745 (58.2%)	173 (42.9%)	
Yes	375 (20.6%)	129 (30.8%)		535 (41.8%)	230 (57.1%)	
Use of antihypertensive agents			**<0.001**			**0.001**
No	38 (2.1%)	51 (12.2%)		14 (1.1%)	14 (3.5%)	
ACEIs	251 (13.8%)	43 (10.3%)		166 (13.0%)	50 (12.4%)	
ARBs	836 (45.9%)	156 (37.2%)		594 (46.4%)	145 (36.0%)	
Beta blockers	612 (33.6%)	152 (36.3%)		457 (35.7%)	179 (44.4%)	
CCBs	64 (3.5%)	8 (1.9%)		39 (3.0%)	11 (2.7%)	
Diuretics	20 (1.1%)	9 (2.1%)		10 (0.8%)	4 (1.0%)	

The bold value represents that the *p* < 0.05, and its corresponding mean ± standard deviation in table 1-4 or adjusted OR (95%CI) in figure 1.

Data are given as percentages or mean ± SD, unless otherwise indicated. HUA, hyperuricemia. ACEIs, angiotensin converting enzyme inhibitors; ARB, angiotensin receptor blockers; CCBs, calcium channel blockers.

**Table 4:** Cardiometabolic profiles in hypertensive adults with and without HUA.

Characteristic	18–59 years old			≥60 years old		
	non-HUA	HUA	*p* value	non-HUA	HUA	*p* value
SBP (mmHg)	**(151.7 ± 8.9)**	**(152.8 ± 8.9)**	**0.028**	**(150.4 ± 7.7)**	**(151.5 ± 7.6)**	**0.016**
DBP (mmHg)	(88.0 ± 8.2)	(87.6 ± 8.1)	0.397	(86.2 ± 8.4)	(86.3 ± 8.5)	0.801
WC (cm)	(85.7 ± 12.9)	(86.8 ± 15.2)	0.175	(89.3 ± 16.8)	(89.4 ± 17.8)	0.889
BMI (kg/m^2^)	**(24.9 ± 2.5)**	**(25.3 ± 2.8)**	**0** **.** **001**	(25.1 ± 2.6)	(25.1 ± 2.9)	0.660
UA (umol/L)	**(292.1 ± 68.3)**	**(459.3 ± 70.9)**	**<0** **.** **001**	**(288.2 ± 78.0)**	**(453.1 ± 71.0)**	**<0** **.** **001**
FBG (mmol/L)	**(5.6 ± 1.1)**	**(5.9 ± 1.4)**	**0** **.** **001**	**(5.9 ± 1.4)**	**(6.1 ± 1.2)**	**0** **.** **003**
TC (mmol/L)	(4.5 ± 1.0)	(4.6 ± 1.4)	0.185	**(4.5 ± 1.0)**	**(4.9 ± 1.4)**	**<0** **.** **001**
TG (mmol/L)	**(1.5 ± 0.7)**	**(2.0 ± 1.2)**	**<0** **.** **001**	**(1.6 ± 0.7)**	**(2.1 ± 1.3)**	**<0** **.** **001**
LDL-C (mmol/L)	**(2.4 ± 0.8)**	**(2.7 ± 1.2)**	**<0.001**	**(2.3 ± 0.9)**	**(2.8 ± 0.9)**	**<0.001**
HDL-C (mmol/L)	**(1.5 ± 0.6)**	**(1.8 ± 1.0)**	**<0.001**	**(1.5 ± 0.6)**	**(1.7 ± 0.8)**	**0.006**
SCr (umol/L)	**(71.2 ± 18.0)**	**(73.6 ± 18.3)**	**0.014**	(72.1 ± 24.8)	(73.6 ± 25.0)	0.309
eGFR (ml/min/1.73m^2^)	**(113.4 ± 50.1)**	**(107.4 ± 38.0)**	**0.021**	(100.8 ± 213.9)	(107.7 ± 311.3)	0.675

The bold value represents that the *p* < 0.05, and its corresponding mean ± standard deviation in table 1-4 or adjusted OR (95%CI) in figure 1.

Data are given as percentages or mean ± SD, unless otherwise indicated. HUA, hyperuricemia; SBP, systolic blood pressure; DBP, diastolic blood pressure; WC, waist circumference; BMI, body mass index; UA, uric acid; FBG, fasting blood glucose; TC, total cholesterol; TG, triglycerides; LDL-C, low density lipoprotein cholesterol; HDL-C, high density lipoprotein cholesterol; SCr, serum creatinine; eGFR, estimated glomerular filtration rate.

The young and middle-aged hypertensive adults with HUA had a higher proportion of females and used aspirin and a lower proportion of used antihypertensive agents than adults without HUA. What's more, the levels of SBP, BMI, UA, FBG, TG, LDL-C, HDL-C, and SCr were higher, and the level of eGFR was lower than adults without HUA.

Similar patterns were observed in elderly adults. Among adults with HUA, the proportion of females and the use of aspirin and the levels of SBP, UA, FBG, TG, LDL-C, and HDL-C were higher, and the proportion used antihypertensive agents was lower than adults without HUA. Unlike the young and middle-aged adults, the adults with HUA had higher TC levels than those without HUA in the elderly group. Moreover, there was no significant difference in BMI, Scr, and eGFR between adults with HUA and without HUA.

### Multivariate logistic regression analysis of HUA

We conducted multivariate logistic regressions to explore the relationship between HUA and other cardiometabolic risk factors in the two groups (Figure 1 and Supplementary Table S1).

Among young and middle-aged adults, HUA was associated with higher BMI (adjusted OR = 1.114, 95% CI: 1.057–1.174), higher FBG (adjusted OR = 1.099, 95% CI: 1.003–1.205), higher TG (adjusted OR = 1.425, 95% CI: 1.247–1.629), higher LDL-C (adjusted OR = 1.171, 95% CI: 1.025–1.337), and lower eGFR (adjusted OR = 0.992, 95% CI: 0.988–0.996), after adjusting for age, sex, education level, use of aspirin, and use of antihypertensive agents.

Among elderly adults, higher SBP (adjusted OR = 1.024, 95% CI: 1.005–1.042), higher TG (adjusted OR = 1.716, 95% CI: 1.466–2.009), and higher LDL-C (adjusted OR = 1.595, 95% CI: 1.366–1.863) were associated with HUA. However, unlike the young and middle-aged adults, BMI, FBG, and eGFR did not seem to relate to HUA among elderly hypertensive adults after adjusting for other potential confounders.

## Discussion

We found that HUA is closely associated with higher blood lipid profiles (containing TG and LDL-C), FBG, BMI, and lower eGFR in young and middle-aged hypertensive adults in Beijing population. In elderly hypertensive adults, HUA was associated with higher SBP, TG, and LDL-C. HUA was associated with more cardiometabolic risk factors in young and middle-aged hypertensive adults than in the elderly.

A series of epidemiological studies (14) have shown the association of HUA with cardiovascular diseases and risk factors like high LDL cholesterol and hypertriglyceridemia (15). A previous study has also shown that younger men with HUA had higher blood pressure (BP; a major risk factor for CVD) than older men (16). It is still unclear why agents that lower serum UA may not lower BP or may lower BP in only a select population (17). Our study finds the association of HUA with HT jointly predisposes young and middle-aged people to more cardiovascular risk factors than elderly people with HT and HUA. The mechanism of how HUA and HT jointly increase the risk of cardiovascular disease is unknown. HUA was shown to cause HT and renal injury in the rat *via* a crystal-independent mechanism, with stimulation of the renin-angiotensin system (RAS) and inhibition of neuronal NO synthase (18). HUA can cause thickening of the preglomerular arteries with smooth muscle cell proliferation and increase renal renin and COX-2 expression (19, 20) resulting in elevation of blood pressure in hyperuricemic rats that was reduced after allopurinol treatment (17). Dysregulation of the immune system by induction of COX-2, interleukin-1β expression and generation of superoxide radical (*O*_2_^−^) by xanthine oxidoreductase (XOR) in HUA (21) and impaired renal function (reduced GFR) (19) together constitute a risk factor for metabolic syndrome. Metabolic syndrome and cardiovascular diseases are epidemiologically and pathophysiologically intertwined.

Several studies have found the difference in association of HUA and other risk indicators between young people and the elderly, but few study has focused on HUA and cardiometabolic risk factors in hypertensive population. Brand et al. (22) found that the association between UA and the risk of coronary heart disease and future diabetes diminished with advanced age. A study by Sundström et al. (23) indicated that the relationship between elevated serum UA and HT wakened with increasing follow-up time by comparing previous cohort studies. A prospective nested Case-control study (10) demonstrated that elevated serum UA was significantly associated with HT in men <60 years after adjusting for other metabolic factors, whereas it was not significantly associated with HT in men ≥60 years. An 8-year prospective cohort study (24) recently suggested that HUA was more strongly associated with HT and hypertriglyceridemia in young adults. In addition, a meta-analysis (12) of 15 cohorts with 99,205 individuals specifically explored the effect of age on the association between UA and CKD in subgroup analyses. The results showed that HUA was significantly associated with CKD among adults younger than 60 years of age (RR = 1.26, *p* value = 0.022). In contrast, no significant association was observed in adults aged 60 years or older (RR = 1.04, *p* value = 0.409).

In this study, we found that HUA is closely associated with more cardiometabolic risk factors in hypertensive young and middle-aged Beijing people than hypertensive elderly. Age-related differences in inflammation and hemostasis response (25) might explain why HUA is closely correlated with more cardiometabolic risk factors in young and middle-aged people, as in younger age, critical illness is associated with a sharp increase in inflammation and hemostasis reactions (25) although along with a quick resolution. On the other hand, HUA-associated activation of RAS and insulin resistance (26, 27), which are pronounced in younger hypertensive people, might contribute to more cardiometabolic disorders in this population. Hypertension in elderly is mainly attributable to increased vascular stiffness, salt sensitivity and reduced renal function (10, 18, 28), and in that stage, hypertension is mainly driven by the kidney, and lowering UA levels is no longer protective (18, 28).

This study has several limitations. First, this study is cross-sectional, the information on correlation of the trajectory of UA and other risk factors with cardiovascular diseases are lacking, and the causal relationship cannot be determined. Second, this study only included the hypertensive population in Beijing, so we should be cautious when extending the conclusion to a broader population. Third, although we have included many demographic and clinical parameters in the analysis, more factors with predictive potential or affecting the correlation may need to be considered, such as lifestyles, and the use of lipid -lowering drugs, especially among young and middle-aged people.

In fact, the causal role of HUA for cardiovascular diseases is yet to be proven. The current convincing evidence for the causal role of UA exists only for gout and nephrolithiasis. More basic and clinical trials are needed to clarify the relationship between HUA and cardiovascular disease, and to determine the benefits of lowering UA levels in preventing or treating cardiovascular diseases. Besides HUA, the levels of testosterone (27) in male, estrogen in female (29) and melatonin (30, 31) might play important roles in cardiovascular diseases and warrants future studies.

## Conclusion

The results of our study suggest that HUA is closely associated with more cardiometabolic disorders in young and middle-aged hypertensive adults in Beijing population. Comprehensive management of HT with HUA is needed in clinical settings.

## Data Availability

The raw data supporting the conclusions of this article will be made available by the authors, without undue reservation.
