# Supplementary material for: Hyperuricemia is associated with more cardiometabolic risk factors in hypertensive younger Chinese adults than in elderly
Source: Front Cardiovasc Med. 2023 Mar 17;10:1133724. doi: 10.3389/fcvm.2023.1133724 (PMC10063877; doi:10.3389/fcvm.2023.1133724)
Supplement: Supplementary file 1 [file Table1.docx]

Supplementary Material

Hyperuricemia is Associated with More Cardiometabolic Risk Factors in Hypertensive Younger Chinese Adults than in Elderly.

**Xiaofeng Su1, Jing Liu1*, Ningling Sun1 and Yong Huo2* On behalf of the SUCCESS investigation group**

*** Correspondence:** Jing Liu: [heartcenter@163.com](mailto:heartcenter@163.com); Yong Huo: huoyong@263.net.cn

# Supplementary Table 1 Multivariate logistic regression analysis of HUA in different age groups

| **Characteristic** | **18-59 years old** | | **≥60 years old** | |
| --- | --- | --- | --- | --- |
|  | **OR(95%CI)** | ***p* value** | **OR(95%CI)** | ***p* value** |
| **Age** | 1.000(0.979,1.021) | 0.979 | 0.993(0.973,1.013) | 0.491 |
| **Female*** | **1.434(1.099,1.871)** | **0.008** | **2.518(1.940,3.268)** | **<0.001** |
| **education** |  | 0.850 |  | 0.089 |
| **Senior high school**** | 1.059(0.714,1.570) | 0.777 | 0.855(0.634,1.154) | 0.306 |
| **Junior college**** | 0.927(0.637,1.351) | 0.695 | 0.660(0.469,0.928) | 0.017 |
| **Undergraduate or above**** | 1.022(0.691,1.513) | 0.912 | 0.668(0.386,1.155) | 0.149 |
| **Use of aspirin** | **1.593(1.226,2.069)** | **<0.001** | **1.646(1.269,2.135)** | **<0.001** |
| **Use of antihypertensive agents** | | **<0.001** |  | **0.004** |
| **ACEIs***** | **0.137(0.076,0.245)** | **<0.001** | **0.320(0.128,0.799)** | **0.015** |
| **ARBs***** | **0.162(0.097,0.271)** | **<0.001** | **0.259(0.109,0.620)** | **0.002** |
| **Beta blockers***** | **0.193(0.115,0.322)** | **<0.001** | **0.373(0.156,0.893)** | **0.027** |
| **CCBs***** | **0.103(0.042,0.254)** | **<0.001** | **0.186(0.061,0.562)** | **0.003** |
| **Diuretics***** | **0.378(0.144,0.993)** | **0.048** | 0.562(0.124,2.558) | 0.456 |
| **SBP** | 1.014(1.000,1.029) | 0.057 | **1.024(1.005,1.042)** | **0.012** |
| **DBP** | 1.007(0.993,1.022) | 0.324 | 1.001(0.986,1.017) | 0.905 |
| **WC** | 1.002(0.992,1.013) | 0.637 | 0.998(0.989,1.008) | 0.761 |
| **BMI** | **1.114(1.057,1.174)** | **<0.001** | 1.049(0.997,1.103) | 0.063 |
| **FBG** | **1.099(1.003,1.205)** | **0.044** | 1.008(0.912,1.114) | 0.878 |
| **TC** | 1.029(0.923,1.148) | 0.602 | 1.122(0.997,1.263) | 0.057 |
| **TG** | **1.425(1.247,1.629)** | **<0.001** | **1.716(1.466,2.009)** | **<0.001** |
| **LDL-C** | **1.171(1.025,1.337)** | **0.020** | **1.595(1.366,1.863)** | **<0.001** |
| **HDL-C** | 1.132(0.952,1.346) | 0.160 | 0.848(0.697,1.033) | 0.102 |
| **eGFR** | **0.992(0.988,0.996)** | **<0.001** | 1.000(1.000,1.001) | 0.542 |

* compared with male; **compared with junior high school or below; ***compared with no use of antihypertensive agent.

HUA, hyperuricemia; OR, odds ratio; CI, confidence interval; ACEIs, angiotensin converting enzyme inhibitors; ARB, angiotensin receptor blockers; CCBs: calcium channel blockers; SBP, systolic blood pressure; DBP, diastolic blood pressure; WC, waist circumference; BMI, body mass index; FBG, fasting blood glucose; TC, total cholesterol; TG, triglycerides; LDL-C, low density lipoprotein cholesterol; HDL-C, high density lipoprotein cholesterol; SCr, serum creatinine; eGFR: estimated glomerular filtration rate.
